# Synthesis, Electron Transport Behavior, and Enhanced Blue Light Stability of Polyfluorene-Poly(Methyl Methacrylate) Diblock Copolymers

**DOI:** 10.3390/mi17040487

**Published:** 2026-04-16

**Authors:** Ruoyu Jiang, Changchun Liu, Jin Cheng, Kenji Ogino

**Affiliations:** 1 Department of Chemical Engineering and Pharmaceutical Engineering, Changzhou Vocational Institute of Engineering, Changzhou 213164, China; ryjiang@czie.edu.cn (R.J.); ccliu@czie.edu.cn (C.L.); 2 Graduate School of Bio-Applications and Systems Engineering, Tokyo University of Agriculture and Technology, 2-24-16 Nakacho, Koganei-shi 184-8588, Tokyo, Japan

**Keywords:** poly(methyl methacrylate), AB-type block copolymer, blue-light stability, electron mobility

## Abstract

Poly(9,9-di-*n*-octylfluorene) (PFO) suffers from interchain aggregation, which degrades its blue spectral stability and charge transport. To address this, a series of rod-coil diblock copolymers (PFO-*b*-PMMAs) with varying poly(methyl methacrylate) (PMMA) chain lengths were synthesized via Steglich coupling. The non-conjugated PMMA blocks act as bulky steric spacers in the solid state, effectively suppressing detrimental PFO aggregation and enhancing pure blue emission stability. Furthermore, moderate PMMA blocks (PFO-*b*-PMMA1 and PFO-*b*-PMMA2) promote favorable *β*-phase formation and ordered crystalline packing. This microstructural optimization yields a maximum electron mobility of 1.98 × 10^−6^ cm^2^/(V·s) for PFO-*b*-PMMA2, markedly higher than the PFO-2 homopolymer (4.13 × 10^−7^ cm^2^/(V·s)). However, an overlong PMMA block (PFO-*b*-PMMA3) introduces excessive steric hindrance (*T*_g_ = 66 °C) that disrupts crystallization, acting as an insulating barrier that reduces mobility. Thus, precisely tuning the non-conjugated block length effectively maximizes both the blue spectral stability and electron transport capabilities of PFO-based materials.

## 1. Introduction

Polymer light-emitting diodes (PLEDs) have generated considerable research interest and are rapidly advancing the development of next-generation flexible displays and large-area solid-state lighting technologies [1,2,3,4,5]. This immense potential is largely attributed to their inherently low manufacturing costs through solution processing, outstanding mechanical flexibility, fast operational response, and remarkable thermal stability. Among the diverse array of conjugated polymers utilized as emitting layers, poly(9,9-di-*n*-octylfluorene) (PFO) stands out as a preeminent candidate for deep-blue emission [6,7,8,9,10]. Its rigid, rod-like biphenyl backbone endows it with an exceptionally high photoluminescence quantum efficiency. Furthermore, the presence of alkyl side chains ensures excellent solubility in common organic solvents, facilitating high-quality, uniform film formation during spin-coating or inkjet printing processes.

Despite these compelling advantages, the commercialization of PFO in practical device applications is severely hindered by two intrinsic limitations. First, the morphological instability of PFO remains a critical challenge. When subjected to prolonged thermal annealing, photo-irradiation, or electrical stress during device operation, the rigid conjugated polymer chains exhibit a strong tendency to tightly aggregate and undergo π–π stacking. This interchain aggregation frequently leads to the formation of excimers. Concurrently, the unshielded C9 positions on the fluorene units are susceptible to localized oxidation, yielding fluorenone defects. These combined degradation pathways introduce a broad, undesirable low-energy green emission band centered around 530 nm, which drastically degrades the spectral purity and overall blue-light stability of the material [11,12]. Second, PFO suffers from intrinsically unbalanced charge carrier transport. The presence of inherent electron traps within the polymer matrix restricts electron mobility compared to hole mobility. This charge imbalance not only shifts the recombination zone closer to the cathode but also triggers Shockley–Read–Hall (SRH) trap-assisted non-radiative recombination. Consequently, a significant portion of injected charge carriers is lost, strictly limiting the macroscopic power efficiency and luminance of the corresponding devices [13,14,15].

To address these interconnected electronic and morphological drawbacks, extensive efforts have been devoted to modifying the molecular architecture of polyfluorenes. Prevailing strategies encompass main-chain copolymerization to disrupt conjugation symmetry, side-chain engineering with bulky substituents to induce steric hindrance, and end-group functionalization [16,17,18]. Among these methodologies, covalently attaching non-conjugated, flexible polymer segments to the termini of rigid PFO chains to construct rod-coil block copolymers has emerged as a highly effective approach. This architectural design successfully regulates the nanoscale morphological structure and interchain packing of the conjugated backbone without fundamentally compromising its intrinsic electronic bandgap [19,20,21]. Building upon this paradigm, recent investigations by our group have systematically explored the end-capping of PFO. We previously established that introducing a solitary flexible coil, such as polystyrene (PSt) or poly(ethylene oxide) (PEO), at a single terminus of the PFO backbone acts as a powerful morphological director [22,23]. These flexible coils were found to intricately modulate the microscopic crystallization kinetics, specifically promoting the formation of the highly ordered *β*-phase conformation, which in turn yielded substantial enhancements in macroscopic electron mobility. More recently, we expanded this concept by synthesizing an AB_2_-type miktoarm block copolymer (PFO-*b*-PMMA_2_) incorporating poly(methyl methacrylate) (PMMA) [24]. PMMA is particularly advantageous due to its excellent optical transparency and significant inherent steric bulk. Our findings verified that the branched PMMA architecture provided sufficient spatial separation to weaken detrimental interchain π–π interactions, thereby remarkably elevating the blue-light spectral stability under stress while concurrently preserving efficient electron transport pathways.

While the complex AB_2_-type miktoarm topology demonstrated undeniable performance benefits, it also introduces significant synthetic and morphological complexities. The linear AB-type diblock structure, serving as the most fundamental and universally recognized macromolecular model, possesses irreplaceable theoretical value. Investigating this simplified linear architecture is essential for achieving a fundamental, bottom-up understanding of how a single flexible PMMA segment regulates the complex chain entanglement, crystalline nucleation, and solid-state aggregation behaviors of a rigid conjugated polyfluorene rod. Motivated by the need to isolate these fundamental structure–property relationships, the present study reports the design and synthesis of a series of novel, linear AB-type diblock copolymers (PFO-*b*-PMMAs) featuring a single linear PMMA segment attached to one end of the PFO chain. This manuscript details the precise synthetic pathway utilized to obtain these linear architectures and provides a systematic investigation into how varying the length of the flexible PMMA segment dictates the thermal transitions, optical absorption and emission characteristics, and thin-film crystalline packing of the PFO backbone. Furthermore, by fabricating and analyzing single-carrier electron-only (EO) devices, we rigorously evaluate the impact of this architectural modification on the trap-limited electron transport behavior. Ultimately, this work seeks to comprehensively elucidate the synergistic mechanisms by which linear PMMA end-group functionalization simultaneously suppresses aggregation to enhance blue-light stability and optimizes microscopic packing to elevate charge carrier mobility, thereby supplying a robust theoretical and experimental framework for the next generation of highly stable, high-performance PLED materials.

## 2. Materials and Methods

### 2.1. Materials

Methyl methacrylate (MMA) was purified via vacuum distillation prior to use. N,N,N’,N′,N″-pentamethyldiethylenetriamine (PMDETA), N,N′-dicyclohexylcarbodiimide (DCC), and 4-dimethylaminopyridine (DMAP) were commercial products and used as received. Dry tetrahydrofuran (THF) was refluxed over sodium metal wire in the presence of benzophenone as a visual indicator. The distillation of THF was carried out and collected only after the solvent mixture exhibited a persistent, deep blue ketyl radical coloration, confirming the complete elimination of trace water and dissolved oxygen. Dry dichloromethane (DCM) was dried by prolonged refluxing over calcium hydride (CaH_2_), followed by careful fractional distillation. All freshly distilled solvents were immediately stored and manipulated under a high-purity nitrogen atmosphere. Mono-carboxylic acid-terminated poly(methyl methacrylate) (PMMA-COOH) was synthesized via atom transfer radical polymerization (ATRP) using an acid-functionalized initiator, following a procedure analogous to our previously reported synthesis of PSt-COOH [21,22].

### 2.2. Synthesis of Hydroxyl End-Functionalized Polyfluorene (PFO-2)

The tert-butyldimethylsiloxyethoxy end-capped PFO (PFO-1) and the subsequent hydroxyl end-functionalized polyfluorene (PFO-2) were synthesized according to the procedures previously reported by our research group [22,23]. Briefly, PFO-1 was prepared via the Suzuki–Miyaura cross-coupling reaction. Subsequently, the protective group of PFO-1 was removed using HF/pyridine to afford the single hydroxyl end-capped PFO-2.

### 2.3. Synthesis of Mono-Carboxylic Acid-Terminated Poly(Methyl Methacrylate) (PMMA-COOH)

CuBr (0.86 g, 6.0 mmol), CuBr_2_ (0.066 g, 0.3 mmol), PMDETA (1.26 mL, 3.0 mmol), 2-bromopropionic acid tert-butyl ester (0.94 mL, 6.0 mmol), deoxygenated toluene (30 mL), and distilled methyl methacrylate were charged into a round-bottomed flask. After deoxygenation via four freeze–pump–thaw cycles, the mixture was stirred at 50 °C for 1 h under nitrogen. The cooled mixture was diluted with toluene, filtered through active alumina, concentrated, and precipitated in 1:1 (*v*/*v*) methanol/water mixture to yield the tert-butyl ester-terminated PMMA intermediate.

Subsequently, this intermediate (1 mmol), *p*-toluenesulfonic acid monohydrate (1.72 g, 10 mmol), and anhydrous dioxane (20 mL) were added to a nitrogen-purged flask. The mixture was stirred at 95 °C for 24 h. Finally, the resulting solution was precipitated into a 1:1 (*v*/*v*) methanol/water mixture to afford the desired PMMA-COOH as a white solid.

### 2.4. Synthesis of AB-Type PFO-b-PMMA

The linear AB-type block copolymer, PFO-*b*-PMMA, was synthesized via a mild Steglich esterification coupling reaction, following the exact methodology we previously developed for PFO-*b*-PSt and PFO-*b*-PEO copolymers [22,23]. Briefly, PFO-2, PMMA-COOH, DCC, and DMAP were placed in a reaction flask. Dry dichloromethane was added as the solvent under a nitrogen atmosphere. The mixture was stirred continuously at room temperature for 24 h. After the reaction, the resulting solution was evaporated to remove the solvent, and the residue was crushed by stirring overnight with acetone. Finally, the filtrated solid was purified via Soxhlet extraction with acetone to effectively remove the unreacted PMMA homopolymer and coupling byproducts, affording the pure linear PFO-*b*-PMMA block copolymer.

### 2.5. Characterizations

To determine the chemical structures, proton nuclear magnetic resonance spectra (^1^H-NMR) spectroscopy was performed on a JEOL ALPHA300 spectrometer (JEOL Ltd., Tokyo, Japan) operating at 300 MHz (25 °C). All NMR samples were dissolved in deuterated chloroform (CDCl_3_) containing tetramethylsilane as the zero-point reference.

Molecular weight distributions, including number-average molecular weights (*M*_n_) and polydispersity indices (PDIs), were assessed via gel permeation chromatography (GPC; JASCO RI-2031, Jasco Corporation, Tokyo, Japan). The GPC system utilized chloroform as the mobile phase and was calibrated against narrowly distributed polystyrene standards.

Thermal transitions, specifically the glass transition temperatures (*T*_g_), were captured with a Rigaku DSC-8230 calorimeter (Rigaku, Tokyo, Japan). These scans were conducted in a flowing nitrogen environment applying a constant temperature ramp of 10 °C/min.

For optical characterization, JASCO V-570 (Jasco Corporation, Tokyo, Japan) and JASCO FP-6500 (Jasco Corporation, Tokyo, Japan) were employed to collect ultraviolet–visible (UV–Vis, Jasco Corporation, Tokyo, Japan) and photoluminescence (PL, Jasco Corporation, Tokyo, Japan, *λ*_ex_ = 380 nm) spectra, respectively. Solid-state films for these measurements were cast onto glass substrates from chlorobenzene solutions (40 mg/mL) via spin-coating at 1000 rpm for 60 s.

Microstructural packing within the films was probed using grazing incidence wide-angle X-ray diffraction (GIWAXD) on a RIGAKU SmartLab system (Rigaku, Tokyo, Japan), while a BRUKER Dektak XT-S profilometer (BRUKER, Billerica, MA, USA) was utilized to confirm film thicknesses.

### 2.6. EO Device Fabrication

To evaluate charge transport capabilities, single-carrier electron-only (EO) devices were assembled utilizing patterned indium tin oxide (ITO)-coated glass (sheet resistance ~10 Ω/sq). Prior to deposition, these substrates underwent a rigorous ultrasonic cleaning protocol, first in an alkaline detergent and subsequently in *2*-propanol, followed by thorough drying under a stream of pure nitrogen. The targeted device architecture was ITO/Al (50 nm)/polymer film (150 nm)/LiF (0.5 nm)/Al (100 nm). Initially, a 50 nm aluminum electron-injection layer was thermally evaporated directly onto the clean ITO. Subsequently, the active polymer layers were deposited via spin-casting from chlorobenzene. The device structure was completed by the sequential thermal evaporation of a thin lithium fluoride (LiF) buffer layer (0.5 nm) and an aluminum capping electrode (100 nm) under a high vacuum environment (~3.0 × 10^−4^ Pa). Device performance was characterized by recording the dark current–voltage (*J*–*V*) profiles utilizing a Keithley 2400 source-measure unit (Keithley Instruments, Cleveland, OH, USA).

## 3. Results and Discussion

### 3.1. Synthesis and Characterization

**Scheme 1:**
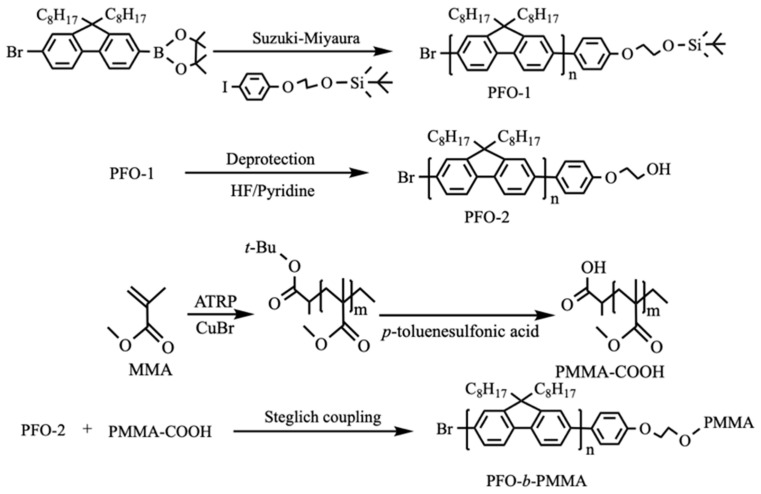
Synthesis route.

In Scheme 1, we show the synthesis routes of PFO-2 and PFO-*b*-PMMAs.

The precursor PFO-1 was synthesized via Suzuki–Miyaura cross-coupling, followed by deprotection using HF/pyridine to yield hydroxyl-terminated PFO-2. In parallel, carboxyl-terminated PMMA (PMMA-COOH) was prepared via ATRP. The target rod-coil block copolymers, PFO-*b*-PMMAs, were successfully synthesized via Steglich coupling between PFO-2 and PMMA-COOH.

The *M*_n_ and PDIs characterized by GPC (Figure S5) and ^1^H-NMR (Figures S1–S4) are summarized in Table 1. The starting PFO-2 exhibited an *M*_n_ of 7468 g/mol (by GPC) with a PDI of 2.11. To study the effect of the flexible chain length, three copolymers with varying PMMA block lengths (*M*_n,PMMA-section_ = 1030, 1990, and 3120 g/mol) were synthesized. As the PMMA segment elongated, the total *M*_n_ of the block copolymers increased accordingly, with GPC results matching the ^1^H-NMR calculations. Furthermore, the PDIs of the PFO-*b*-PMMAs decreased to 1.84–2.02, indicating a well-controlled molecular weight distribution.

### 3.2. Thermal Properties

**Figure 1:**
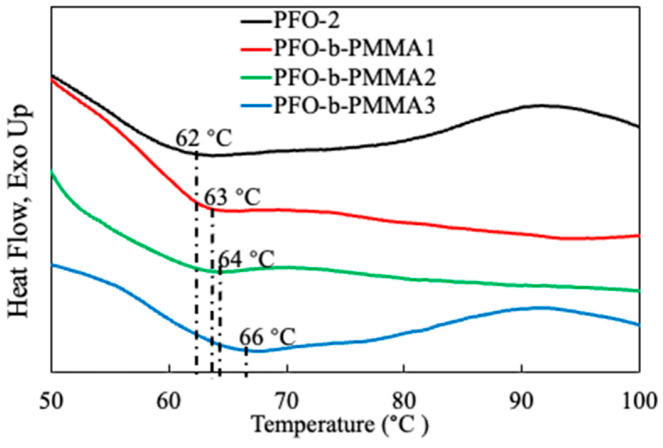
DSC thermograms for PFO-2 and PFO-*b*-PMMAs.

As depicted in Figure 1, the *T*_g_ of the PFO-2 homopolymer was 62 °C. Upon incorporating and elongating the PMMA block, the *T*_g_ progressively increased: 63 °C for PFO-*b*-PMMA1, 64 °C for PFO-*b*-PMMA2, and 66 °C for PFO-*b*-PMMA3. This upward trend is attributed to the increased steric hindrance and polar interactions introduced by the rigid PMMA segments. The PMMA blocks act as anchors that restrict the free segmental motion of the adjacent PFO chains. Consequently, more thermal energy is required to initiate macroscopic chain movement, thereby shifting the *T*_g_ to higher temperatures.

Furthermore, the broad exothermic peak around 90 °C observed for both PFO-2 and PFO-*b*-PMMA3 is attributed to the cold crystallization of the PFO backbones. Unlike PFO-*b*-PMMA1 and 2, which form ordered crystalline structures initially, the excessive steric hindrance from the overlong PMMA block in PFO-*b*-PMMA3 largely suppresses initial crystallization (consistent with the weakened GIWAXD peaks).

### 3.3. Optical Properties

**Figure 2:**
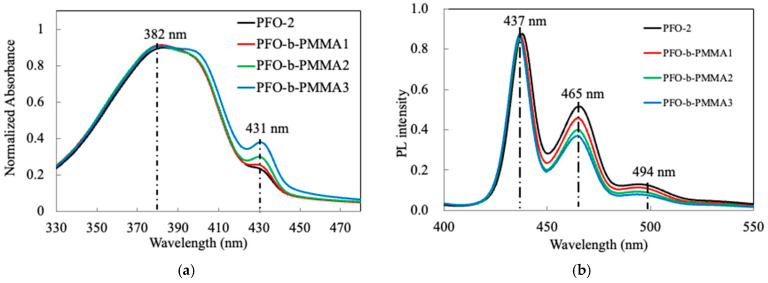
(**a**) UV–Vis and (**b**) PL spectra of PFO-2 and PFO-*b*-PMMAs.

The optical properties of the polymers were evaluated using UV–Vis and PL spectroscopy in the solid film state (Figure 2). As shown in the UV-–Vis spectra (Figure 2a), all polymers exhibited a primary absorption peak around 382 nm. Notably, a characteristic *β*-phase absorption shoulder emerged at 431 nm, accompanied by another observable band at approximately 395 nm (especially for PFO-*b*-PMMA3). This feature arises from the superposition of the 0-1 vibronic transition of the ordered PFO segments (typically around 405 nm) and the broad absorption tail of the amorphous phase. The emergence of these well-resolved vibronic structures (the 0-0 transition at 431 nm and its accompanying higher-energy vibronic bands) indicates that the PMMA segment promotes a more regular, planar PFO backbone conformation in the solid state [25,26,27,28].

Crucially, the film PL spectra (Figure 2b) demonstrated the enhanced blue light stability of the block copolymers. While all polymers showed a main emission peak at 437 nm (associated with the 0-0 purely electronic transition of the π–π* excited state), along with its vibronic replicas at 465 nm (0-1 transition) and 494 nm (0-2 transition) [29,30], the PFO-*b*-PMMAs exhibited suppressed emission intensities in the long-wavelength regions compared to the PFO-2 homopolymer. Consistent with the DSC results, which demonstrated that the rigid PMMA blocks restrict segmental motion, these non-conjugated blocks act as bulky steric spacers in the solid state. They effectively weaken detrimental interchain interactions and suppress aggregation between the PFO backbones [18,31]. Because tight interchain aggregation is the primary trigger for stress-induced green emission defects (e.g., fluorenone and excimers), intrinsically hindering this aggregation fundamentally cuts off the degradation pathways. Thus, this microstructural regulation successfully preserves pure blue emission and effectively enhances the overall blue light stability of the copolymers [32].

### 3.4. Crystalline Structure Analysis

**Figure 3:**
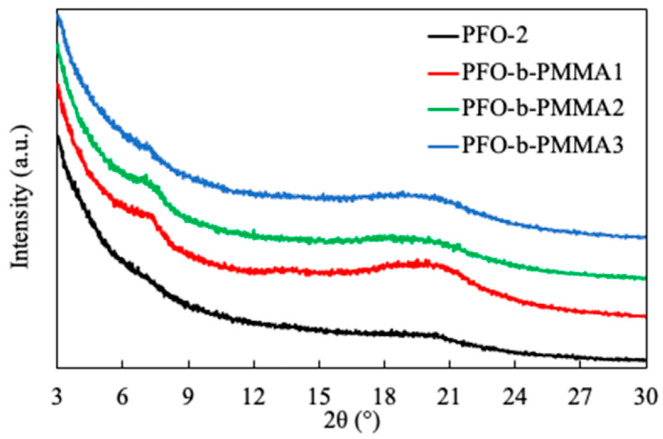
GIWAXD profiles were obtained with out-of-plane geometry for thin films of PFO-2 and PFO-*b*-PMMAs fabricated with chlorobenzene.

The crystalline structures of the polymer films were investigated using X-ray diffraction. As shown in Figure 3, the PFO-2 homopolymer exhibited a relatively weak, nearly amorphous profile. In contrast, the diblock copolymers with appropriately sized PMMA segments (PFO-*b*-PMMA1 and PFO-*b*-PMMA2) displayed distinct diffraction peaks. The peak at approximately 2θ ≈ 20° corresponds to the π–π stacking distances of the conjugated backbones. The peak at 2θ ≈ 7.5° is attributed to lamellar-patterned d-spacings [33]. This demonstrates that the introduction of moderately sized PMMA blocks facilitates the orderly packing and crystallization of the PFO chains.

However, for PFO-*b*-PMMA3 containing the longest PMMA block, these diffraction peaks became noticeably weaker. The presence of an overlong amorphous PMMA chain introduces substantial steric hindrance, which inhibits the extensive crystallite formation of the PFO backbones. This structural evolution aligns perfectly with the previously discussed thermal and optical properties, confirming that optimal block lengths are crucial for regulating the solid-state morphology without disrupting the crystallization entirely.

### 3.5. Electron-Transporting Properties

By analyzing the dark current density–voltage (*J*–*V*) responses within the space-charge-limited current (SCLC) region, the electron mobility (*μ_e_*) for each material was derived using the standard Mott–Gurney relationship (Equation (1)).

(1) $$J=\frac{9}{8}\varepsilon_{r}\varepsilon_{0}\mu_{e}\frac{V^{2}}{L^{3}}$$

In this expression, *J* represents the measured current density, *L* is the precise thickness of the polymer active layer, and *V* defines the effective applied voltage. The parameters *ε*_0_ and *ε_r_* correspond to the vacuum permittivity and the assumed relative dielectric constant of the conjugated polymer (taken as 3.5), respectively.

During data fitting, the voltage *V* was corrected for a 12 Ω series resistance. Furthermore, the built-in potential was omitted from the calculation due to the symmetric nature of the work functions of the contacting electrodes [34].

**Figure 4:**
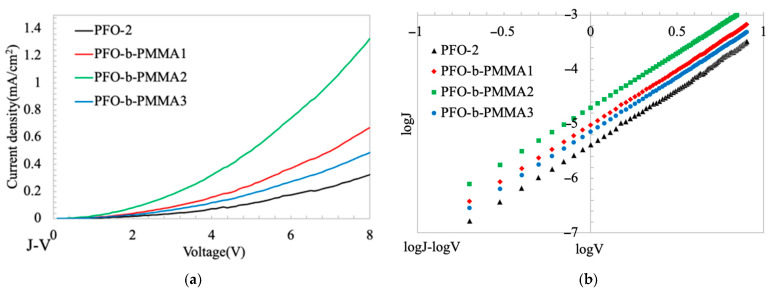
(**a**) Current density–voltage characteristics for electron-transporting devices based on PFO-2 and PFO-*b*-PMMAs; (**b**) double logarithmic plots characteristics for electron-transporting devices based on PFO-2 and PFO-*b*-PMMAs. Films were fabricated via the same procedure as UV–Vis on 3.0 cm × 3.0 cm ITO substrates.

The electron transport behavior is depicted in Figure 4 and Table 2. The electron mobility of the PFO-2 homopolymer was 4.13 × 10^−7^ cm^2^/(V·s). Upon introducing PMMA blocks, the mobility showed an initial increase followed by a decrease: it rose to 9.55 × 10^−7^ and 1.98 × 10^−6^ cm^2^/(V·s) for PFO-*b*-PMMA1 and PFO-*b*-PMMA2, respectively, before dropping to 7.16 × 10^−7^ cm^2^/(V·s) for PFO-*b*-PMMA3.

This trend strongly aligns with the thermal and microstructural evolution of the films. As firmly evidenced by the GIWAXD profiles, the moderate non-conjugated PMMA blocks (PMMA1 and PMMA2) act as steric spacers. They effectively suppress detrimental interchain aggregation to preserve blue light stability while simultaneously promoting *β*-phase formation and highly ordered π–π crystalline packing. This structural optimization significantly enhances the interchain electron hopping efficiency. Conversely, the overlong PMMA block in PFO-*b*-PMMA3 introduces excessive steric hindrance (corresponding to the highest *T*_g_ of 66 °C), acting as an insulating barrier that severely disrupts the extensive crystallization of the PFO backbones. Therefore, an optimal PMMA block length maximizes electron transport capability while enhancing the blue spectral stability of the material.

## 4. Conclusions

A series of rod-coil diblock copolymers, PFO-*b*-PMMAs, with varying PMMA chain lengths were successfully synthesized via Steglich coupling. The non-conjugated PMMA blocks act as bulky steric spacers in the solid state, effectively weakening detrimental interchain aggregation of the rigid PFO backbones. This structural regulation successfully suppresses green defect emissions and significantly enhances the pure blue spectral stability of the materials.

Furthermore, incorporating moderate PMMA block lengths (PFO-*b*-PMMA1 and PFO-*b*-PMMA2) promotes favorable *β*-phase conformation and highly ordered crystalline packing. This microstructural improvement facilitates efficient interchain electron hopping, yielding a maximum electron mobility of 1.98 × 10^−6^ cm^2^/(V·s) for PFO-*b*-PMMA2, markedly higher than that of the PFO-2 homopolymer (4.13 × 10^−7^ cm^2^/(V·s)). However, an overlong PMMA block (PFO-*b*-PMMA3) introduces excessive steric hindrance (*T*_g_ = 66 °C), which acts as an insulating barrier that disrupts PFO crystallization and subsequently reduces electron mobility.

In summary, precisely tuning the length of the non-conjugated flexible block is an effective strategy to optimize the blue light stability of polyfluorene-based materials without sacrificing, and even enhancing their electron transport capabilities.

## Figures and Tables

**Table 1:** Characteristics of PFO-2 and PFO-*b*-PMMAs.

Polymer	*M*_n,PMMA-section_ (g/mol) *^a^*	*M*_n,PFO-_*_b_*_-PMMA_ (g/mol)		PDI *^a^*	PMMA *^b^* (wt%)	Yields
PFO-2	-	7468 ***^a^***	7760 ***^b^***	2.11	-	82%
PFO-*b*-PMMA1	1030	8568 ***^a^***	8790 ***^b^***	2.02	12	75%
PFO-*b*-PMMA2	1990	9491 ***^a^***	9750 ***^b^***	1.97	20	66%
PFO-*b*-PMMA3	3120	10,581 ***^a^***	10,880 ***^b^***	1.84	34	61%

***^a^*** Determined via GPC. ***^b^*** Determined via ^1^H-NMR.

**Table 2:** Electron mobility of PFO-2 and PFO-*b*-PMMAs.

Polymer	Electron Mobility (cm^2^/Vs) *^a^*
PFO-2	4.13 × 10^−7^
PFO-*b*-PMMA1	9.55 × 10^−7^
PFO-*b*-PMMA2	1.98 × 10^−6^
PFO-*b*-PMMA3	7.16 × 10^−7^

***^a^*** Average value determined for four devices.

## Data Availability

The original contributions presented in this study are included in the article/Supplementary Material. Further inquiries can be directed to the corresponding authors.

## Supplementary Materials

The following supporting information can be downloaded at: https://www.mdpi.com/article/10.3390/mi17040487/s1, Figure S1: ^1^H-NMR spectrum of PFO-2; Figure S2: ^1^H-NMR spectrum of PFO-*b*-PMMA1; Figure S3: ^1^H-NMR spectrum of PFO-*b*-PMMA2; Figure S4: ^1^H-NMR spectrum of PFO-*b*-PMMA3; Figure S5: GPC plots of PFO-2 and PFO-*b*-PMMAs.

## References

[1] Burroughes J.H., Bradley D.D.C., Brown A.R., Marks R.N., Mackay K., Friend R.H., Burns P.L., Holmes A.B. (1990). Light-emitting diodes based on conjugated polymers. Nature.

[2] Friend R.H., Gymer R.W., Holmes A.B., Burroughes J.H., Marks R.N., Taliani C., Bradley D.D.C., Dos Santos D.A., Brédas J.L., Lögdlund M. (1999). Electroluminescence in conjugated polymers. Nature.

[3] Zhuo Z., Ni M., Yu N., Zheng Y., Lin Y., Yang J., Sun L., Wang L., Bai L., Chen W. (2024). Intrinsically stretchable fully π-conjugated polymer film via fluid conjugated molecular external-plasticizing for flexible light-emitting diodes. Nat. Commun..

[4] Ma J., Xu M., Zhuo Z., Wang K., Li Q., Li H., Feng Q., Chen W., Yu N., Li M. (2024). Plasticizer design principle of “Like Dissolves Like”: Semiconductor fluid plasticized stretchable fully π-conjugated polymers films for uniform large-area and flexible deep-blue polymer light-emitting diodes. Adv. Mater..

[5] Wan Y., Yuan H.T., Xu Q., Liu F., Cai N., Xue Q., Li X.C., Lai W.Y. (2025). Intrinsically stretchable electroluminescent elastomers based on fully conjugated backbones for organic light-emitting diodes. Macromolecules.

[6] Pei Q., Yang Y. (1996). Efficient photoluminescence and electroluminescence from a soluble polyfluorene. J. Am. Chem. Soc..

[7] Grice A.W., Bradley D.D.C., Bernius M.T., Inbasekaran M., Wu W.W., Woo E.P. (1998). High brightness and efficiency blue light-emitting polymer diodes. Appl. Phys. Lett..

[8] Lu H., Chang C.H., Wu B.R., Wu N.C., Liang J.Z., Dai C.A., Yang A.C.M. (2022). Reaching nearly 100% quantum efficiencies in thin solid films of semiconducting polymers via molecular confinements under large segmental stresses. ACS Nano.

[9] Pidluzhna A., Vembris A., Grzibovskis R., Zommere M.A., Bezvikonnyi O., Simokaitiene J., Baronaite M., Volyniuk D., Grazulevicius J.V., Ali A. (2024). The effect of molecular structure on the properties of fluorene derivatives for OLED applications. Molecules.

[10] Xu Z., Wang L., Wang Y., Niu Z., Liu Y., Wang W., Li S. (2025). Optimized dual-band amplified spontaneous emission properties of PFO and BUBD-1 blend films based on AgNPs doped buffer layers. Opt. Laser Technol..

[11] Gong X., Iyer P.K., Moses D., Bazan G.C., Heeger A.J., Xiao S.S. (2003). Stabilized blue emission from polyfluorene-based light-emitting diodes: Elimination of fluorenone defects. Adv. Funct. Mater..

[12] Nakamura T., Sharma D.K., Hirata S., Vacha M. (2018). Intrachain aggregates as the origin of green emission in polyfluorene studied on ensemble and single-chain level. J. Phys. Chem. C.

[13] Kunz A., Bloma P.W.M., Michels J.J. (2017). Charge carrier trapping controlled by polymer blend phase dynamics. J. Mater. Chem. C.

[14] Abbaszadeh D., Kunz A., Kotadiya N.B., Mondal A., Andrienko D., Michels J.J., Wetzelaer G.A.H., Blom P.W.M. (2019). Electron trapping in conjugated polymers. Chem. Mater..

[15] Khodabakhshi E., Blom P.W.M., Michels J.J. (2019). Efficiency enhancement of polyfluorene: Polystyrene blend light-emitting diodes by simultaneous trap dilution and *β*-phase formation. Appl. Phys. Lett..

[16] Donley C.L., Zaumseil J., Andreasen J.W., Nielsen M.M., Sirringhaus H., Friend R.H., Kim J. (2005). Effects of packing structure on the optoelectronic and charge transport properties in poly(9,9-di-*n*-octylfluorene-alt-benzothiadiazole). J. Am. Chem. Soc..

[17] Bright D.W., Dias F.B., Galbrecht F., Scherf U., Monkman A.P. (2009). The influence of alkyl-chain length on beta-phase formation in polyfluorenes. Adv. Funct. Mater..

[18] Li J., Wang J., Zhou Y., Luo Z. (2014). Synthesis and characterization of polyfluorene-based photoelectric materials: The effect of coil segment on the spectral stability. RSC Adv..

[19] Hsieh H., Hung C., Watanabe K., Chen J., Chiu Y., Isono T., Chiang Y., Reghu R.R., Satoh T., Chen W. (2018). Unraveling the stress effects on the optical properties of stretchable rod-coil polyfluorene-poly (*n*-butyl acrylate) block copolymer thin films. Polym. Chem..

[20] Ahn H.H., Jang S.H., Hwang B., Yoon J., Kim S.Y., Noh S.J., Han J.Y., Kwon Y.K. (2013). Synthesis and self-assembly of a series of π-conjugated triblock copolymers containing polyfluorene and poly (ethylene oxide). Polymer.

[21] Cheng J., Kanehashi S., Ogino K. (2022). Synthesis and electron transporting properties of triblock copolymers consisting of polyfluorene and polystyrene. Jpn. J. Appl. Phys..

[22] Cheng J., Jiang R., Shan Y., Sun H., Kanehashi S., Ogino K. (2024). Synthesis and electron transporting properties of diblock copolymers consisting of polyfluorene and polystyrene. Materials.

[23] Cheng J., Jiang R., Shan Y., Sun H., Kanehashi S., Ogino K. (2024). Polyfluorene-poly(ethylene oxide) diblock copolymers: Synthesis and electron transport behavior. RSC Adv..

[24] Cheng J., Liu C., Shan Y. (2025). Method of introducing polymethylacrylic acid to form AB_2_ type polyfluorene block copolymer and improving its blue light stability. China Synth. Resin Plast..

[25] Peet J., Brocker E., Xu Y., Bazan C.G. (2008). Controlled *β*-phase formation in poly(9,9-di-*n*-octylfluorene) by processing with alkyl additives. Adv. Mater..

[26] Ingle J.D., Crouch S.R. (1988). Spectrochemical Analysis.

[27] Zhang X., Hu Q., Lin J., Lei Z., Guo X., Xie L., Lai W., Huang W. (2013). Efficient and stable deep blue polymer light-emitting devices based on *β*-phase poly (9, 9-dioctylfluorene). Appl. Phys. Lett..

[28] Sun L., Wang S., Zheng Y., Chen W., Li M., Yu N., Wang Y., Yang J., Xu Y., Sun N. (2022). Poly(diarylfluorene) deep-blue polymer light-emitting diodes based on submicrometer-scale morphological films induced by trace *β*-conformation. Macromolecules.

[29] Zhang Q., Wang P.-I., Ong G.L., Tan S.H., Tan Z.W., Hii Y.H., Wong Y.L., Cheah K.S., Yap S.L., Ong T.S. (2019). Photophysical and electroluminescence characteristics of polyfluorene derivatives with triphenylamine. Polymers.

[30] Kidanu H.T., Lee J.H., Chen C.-T. (2021). Photoluminescence and electroluminescence characterization of high-performance near-infrared emitters based on 1,5-naphthyridin-4-ol-containing heteroleptic platinum(II) complexes. Mater. Adv..

[31] Setayesh S., Grimsdale A.C., Weil T., Enkelmann V., Müllen K., Meghdadi F., List E.J.W., Leising G. (2001). Polyfluorenes with polyphenylene dendron side chains: Toward non-aggregating, light-emitting polymers. J. Am. Chem. Soc..

[32] Chochos C.L., Kallitsis J.K., Gregoriou V.G. (2005). Rod-coil block copolymers incorporating terfluorene segments for stable blue light emission. J. Phys. Chem. B.

[33] Hayashi S., Inagi S., Fuchigami T. (2010). Macrostructural order and optical properties of polyfluorene-based polymer films. Polym. J..

[34] Singh C.R., Gupta G., Lohwasser R., Engmann S., Balko J., Thelakkat M., Thurn-Albrecht T., Hoppe H. (2013). Correlation of charge transport with structural order in highly ordered melt-crystallized poly (3-hexylthiophene) thin films. J. Polym. Sci. Part B Polym. Phys..

