# Supplementary figures and images for: Synthesis, Electron Transport Behavior, and Enhanced Blue Light Stability of Polyfluorene-Poly(Methyl Methacrylate) Diblock Copolymers
Source: Micromachines (Basel). 2026 Apr 16;17(4):487. doi: 10.3390/mi17040487 (PMC13118313; doi:10.3390/mi17040487)

Structure of PFO-2

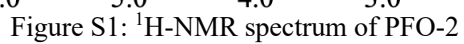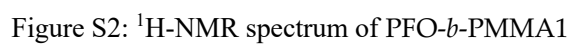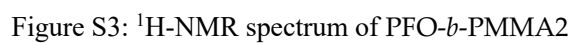

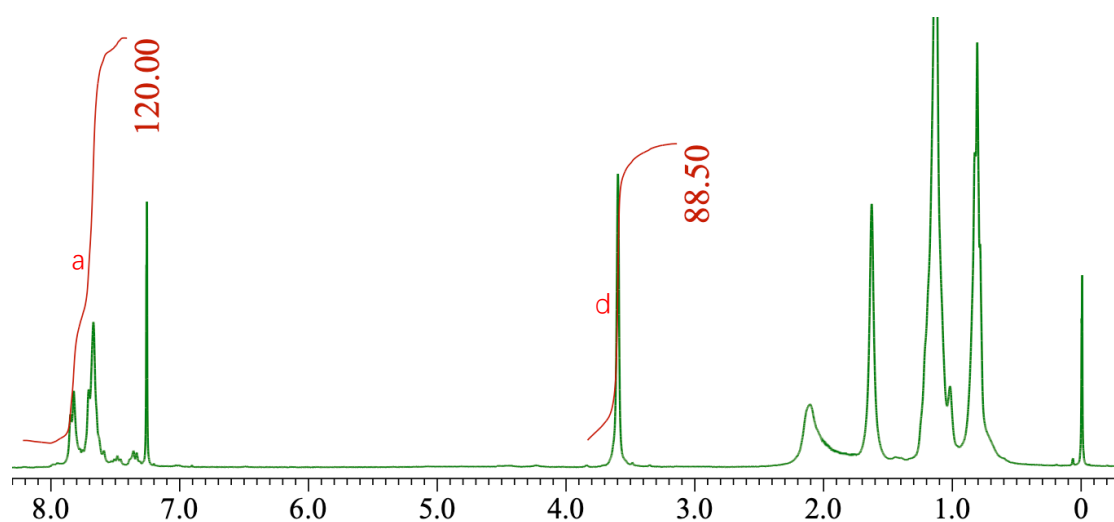

Figure S4:  $^1\text{H}$ -NMR spectrum of PFO-*b*-PMMA3

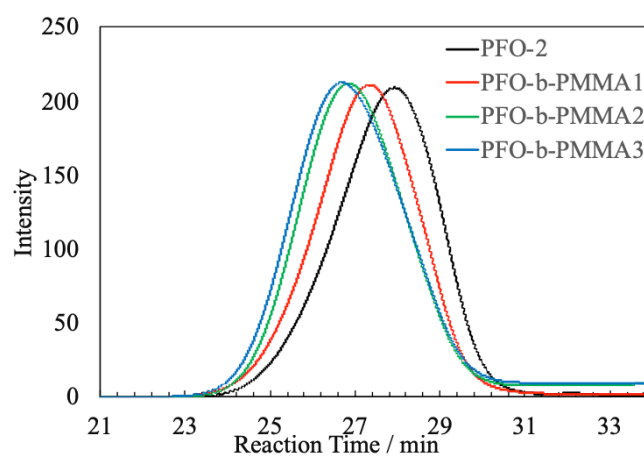

Figure S5: GPC plots of PFO-2 and PFO-*b*-PMMA3

Supplement: Supplementary file 1 [file micromachines-17-00487-s001.zip › micromachines-4241249-supplementary.pdf]
